# Population Genetics and Phylogeography of Galapagos Fur Seals

**DOI:** 10.3389/fgene.2022.725772

**Published:** 2022-05-19

**Authors:** Jaime A. Chaves, Fernando Lopes, Daniela Martínez, Dario F. Cueva, Gabriela I. Gavilanes, Sandro L. Bonatto, Larissa Rosa de Oliveira, Diego Páez-Rosas

**Affiliations:** ^1^ Colegio de Ciencias Biológicas y Ambientales, Universidad San Francisco de Quito USFQ, Diego de Robles y Pampite, Quito, Ecuador; ^2^ Department of Biology, San Francisco State University, San Francisco, CA, United States; ^3^ Escola de Ciências da Saúde e da Vida, Pontifícia Universidade Católica do Rio Grande do Sul, Porto Alegre, Brazil; ^4^ Laboratório de Ecologia de Mamíferos, Universidade do Vale do Rio dos Sinos (UNISINOS), São Leopoldo, Brazil; ^5^ Grupo de Estudos de Mamíferos Aquáticos do Rio Grande do Sul (GEMARS), Torres, Brazil; ^6^ Unidad Técnica Operativa San Cristóbal, Dirección Parque Nacional Galápagos, San Cristobal-Galapagos, Ecuador

**Keywords:** Galapagos fur seal, haplotype, microsatellite, population structure, island

## Abstract

Pinnipeds found across islands provide an ideal opportunity to examine the evolutionary process of population subdivision affected by several mechanisms. Here, we report the genetic consequences of the geographic distribution of rookeries in Galapagos fur seals (GFS: *Arctocephalus galapagoensis*) in creating population structure. We show that rookeries across four islands (nine rookeries) are genetically structured into the following major groups: 1) a western cluster of individuals from Fernandina; 2) a central group from north and east Isabela, Santiago, and Pinta; and possibly, 3) a third cluster in the northeast from Pinta. Furthermore, asymmetric levels of gene flow obtained from eight microsatellites found migration from west Isabela to Fernandina islands (number of migrants *Nm* = 1), with imperceptible *Nm* in any other direction. Our findings suggest that the marked structuring of populations recovered in GFS is likely related to an interplay between long-term site fidelity and long-distance migration in both male and female individuals, probably influenced by varying degrees of marine productivity.

## Introduction

Population structure reflects some degree of discontinuity of its individuals in both space and time. These separations are classically defined by changes in gene frequencies (Wright, 1949) and could be the result of several factors, including interactions between the landscape and microevolutionary processes (e.g., gene flow, genetic drift, and selection) (Manel et al., 2003). Disentangling the relative contributions of such mechanisms can be problematic, but one productive approach involves the detection of genetic discontinuities and correlating these with landscape features. Heterogenous landscapes with naturally fragmented populations can be used to directly assess the role of geographical factors in population divergence (Manel et al., 2003). Habitat islands are discrete, and their attributes provide an independent way to estimate relative levels of migration by providing fixed dispersal distances among populations. Nevertheless, these efforts become challenging with the interplay of behavioral and ecological factors in contributing to the geographic segregation of populations. These challenges are particularly true in mobile species capable of dispersing over large geographic areas (Haag et al., 2010; Dutton et al., 2014) subject to diverse environmental variables affecting their geographic movement (Roberts and Angermeier, 2007; Heupel and Simpfendorfer, 2008).

Some pinnipeds, for example, limit their geographical ranges to small scales since they require terrestrial habitats for mating and parturition (Boyd, 1991; Hoelzel, 2009a; Hoelzel, 2009b; Montero-Serra et al., 2014), which may contribute to the genetic population structure (Riedman, 1990). However, there are otariid species capable of widespread dispersal movements, mainly conducted by males, resulting in a lack of genetic population structure (Fabiani et al., 2003; Campbell et al., 2008; Lowther et al., 2011; Lopes et al., 2015; Oliveira et al., 2017). In addition, local habitat attributes, such as local food type and abundance, have the potential to segregate populations geographically via habitat preference (Cody, 1981; Louzao et al., 2011; Bjørneraas et al., 2012). Therefore, environmental differences and local habitat preference seem to be key to the understanding the genetic divergence in otariids (Wolf et al., 2008; Wolf and Trillmich, 2008). Thus, looking across multiple discrete landscapes (i.e., several islands) along with microevolutionary analyses (i.e., evolutionary processes within species) could reveal how diverse levels of differentiation affect populations in different ecological and evolutionary contexts.

**FIGURE 1:**
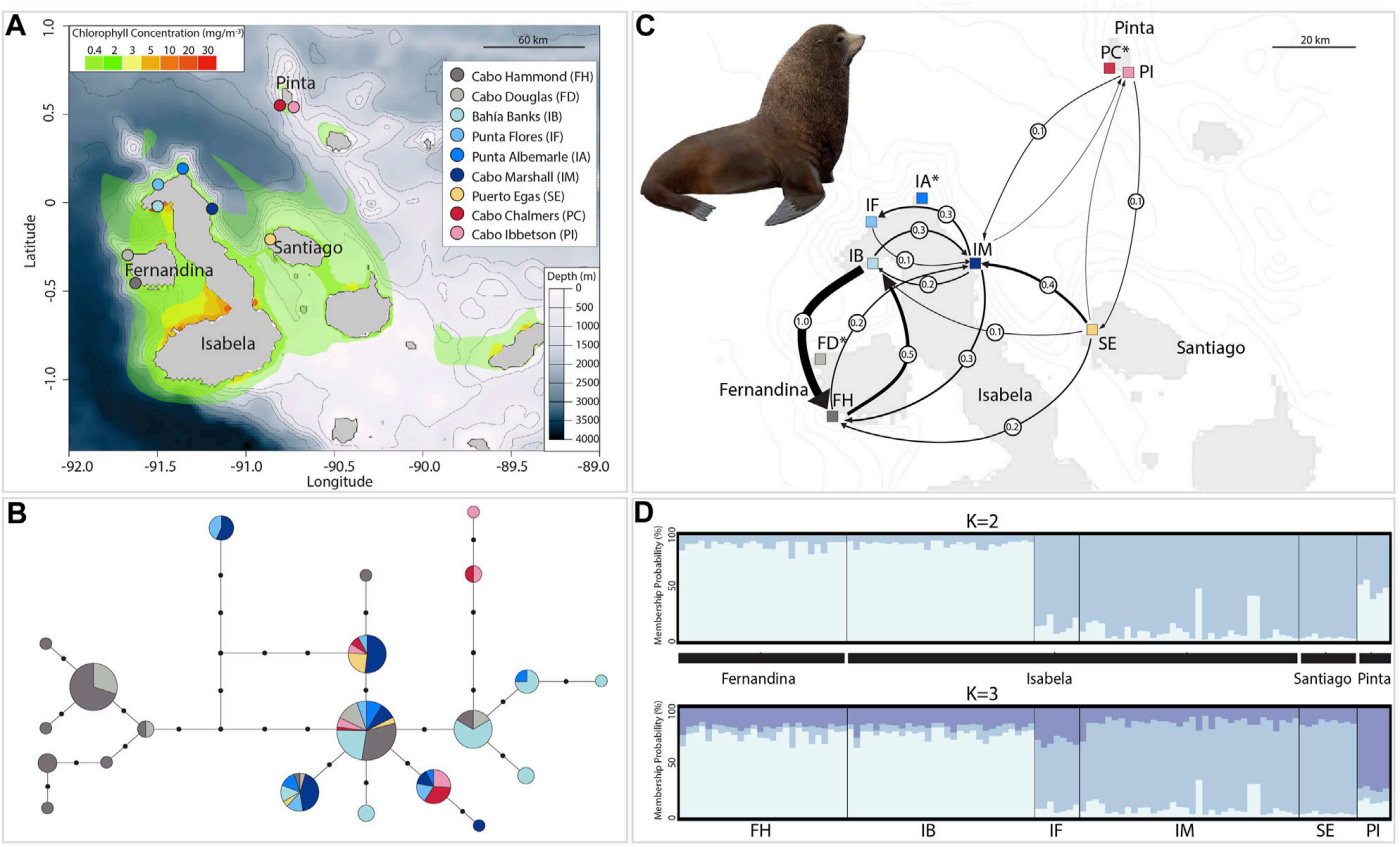
Geographic distribution of genetic diversity, gene flow, and genetic structure in the Galapagos fur seal. **(A)** Map of the Galapagos Islands with the nine breeding rookeries studied (inset) with details about bathymetry shown in meters and chlorophyll-a concentration in mg/m^−3^. **(B)** Mitochondrial haplotype network with circles representing unique *control region* haplotypes; the size of the circles is proportional to the number of individuals sharing that particular haplotype and length of branches (black circles) represent steps between haplotypes (missing haplotypes). Colors correspond to haplotypes found on each of the nine rookeries studied (see inset above). **(C)** Migration estimates based on eight microsatellite markers between six rookeries; black arrows indicate the direction of the gene flow between rookery pairs, and the thickness of each arrow represents the relative amount of migrant exchange (*Nm*: circles over arrows). Arrows with less than 0.1 migrants per generation are not shown (*see* Supplementary Figure S4 for all connections). Rookery names with asterisk were excluded from gene flow analyses. **(D)** STRUCTURE plot of genetic assignment for 110 individuals (vertical lines) of six rookeries based on Bayesian analysis at eight microsatellites showing *K* = 2 suggested by the ΔK method (top) and *K* = 3 after the Puechmille method based on *MedMeaK* or *MedMedK* (bottom). Rookery abbreviations as the inset in **(A)** and black blocks denotes island origin. Illustration reproduced with permission from Lynx Edicions ^©^.

The Galapagos fur seal (GFS) *Arctocephalus galapagoensis* (Heller, 1904) is an endemic species to the Galapagos Islands, and it is considered endangered according to the Red List of Threatened Species by the International Union for Conservation of Nature (IUCN), mainly due to a declining population trend (Trillmich, 2015). This species is the smallest of all otariids and exhibits an unusually restricted geographic range for a pinniped (Churchill et al., 2014). GFS is found in small rookeries throughout the year, with females exhibiting high fidelity to their breeding rookeries (Trillmich, 1987; Páez-Rosas et al., 2012). Most individuals are concentrated on nine rookeries in the western and northern islands of the archipelago (Figure 1A). However, 75% of the population is found on the westernmost island of Fernandina and north of Isabela, with the remaining found in small breeding rookeries towards the east of Santiago and northeast of Pinta (Riofrío-Lazo and Páez-Rosas, 2021). Coincidentally, the western islands are situated in a region of strong upwelling and high productivity (Palacios et al., 2006), leading to a foraging “hotspot” with which endemic otariids are strongly associated (Wolf et al., 2008; Páez-Rosas et al., 2012; Villegas-Amtmann et al., 2013).

GFS, like other otariid species, is capable of widespread dispersal, usually helped by marine currents (Páez-Rosas et al., 2017), which results in high levels of gene flow, which may affect their genetic diversity among populations. Lopes et al. (2015) elucidated the population structure of GFS using matrilineal and nuclear markers. They inferred a weak population structure from nuclear markers, probably due to male-mediated gene flow, contrasting with a remarkably strong fine-scale population structure as revealed by analysis of mitochondrial control region (mtDNA). However, the study by Lopes et al. (2015) only included samples from breeding rookeries from the western region (Fernandina and Isabela islands), missing information from six other rookeries, including Santiago and Pinta islands previously not sampled (Figure 1A).

Although protected within the Galapagos region, GFS have suffered from historical and recent population declines. In particular, environmental instability associated with climatic fluctuations such as El Niño–Southern Oscillation (ENSO) and its effects on marine productivity have caused dramatic reductions in GFS in the last four decades (Páez-Rosas et al., 2021; Riofrío-Lazo and Páez-Rosas, 2021). The strength, length, and frequency of ENSO are variable and appear to be increasing as result of global warming (Chavez et al., 2003; Pennington et al., 2006), and these climatic variations could impact GFS in the near future. In this context, we believe that a new and comprehensive evaluation of the population structure is warranted, including a study of the species distribution in the framework of environmental factors that impact population structure.

The present study updates the population genetics and demographic information of GFS throughout its entire geographic distribution (four islands and nine breeding sites). In particular, we assess the influence of breeding site fidelity and migration using both female-mediated markers (mtDNA) and biparentally inherited markers (microsatellites). In this context, we provide a new and comprehensive evaluation of the effects of behavioral variables (i.e., sex-biased dispersal) impacting the movement of individuals and how landscapes shape functional connectivity between all known populations.

## Methods

### Sampling Design and DNA Extraction

Two molecular marker datasets were analyzed to assess GFS population structure. In both cases, published sequences and genotypes obtained from the study by Lopes et al. (2015) were integrated with genetic data from 69 new samples obtained in 2018. All samples were collected from pups, whose sex was determined on site. Tissues were collected using piglet ear notch pliers (Majluf and Goebel, 1992) from the interdigital membrane of the hind flippers (tissue ∼0.5 cm³). All animals were captured on land with the aid of Galapagos National Park personnel. The samples were stored in 70% ethanol, and genomic DNA extractions were performed with standard phenol–chloroform extraction (Sambrook and Russell, 2001) or the DNeasy Tissue Kit (Qiagen), according to the manufacturer’s instructions.

#### Genetic Datasets

##### mtDNA Control Region

This dataset corresponds to nine breeding rookeries from four islands (Figure 1A). The sequences analyzed include our 69 new samples in addition to 87 published sequences (Lopes et al. (2015)) of 219 bp of the mtDNA control region (Supplementary Table S1), resulting in 156 sequences: 1) Fernandina Island: Cabo Hammond (FH) (n = 44) and Cabo Douglas (FD) (n = 14); 2) Isabela Island: Cabo Marshall (IM) (n = 31), Bahía Banks (IB) (n = 30), Punta Flores (IF) (n = 6), and Punta Albemarle (IA) (n = 10); 3) Pinta Island: Cabo Ibbetson (PI) (n = 8) and Cabo Chalmers (PC) (n = 7); and 4) Santiago Island: Puerto Egas (SE) (n = 6).

##### Microsatellite

This dataset corresponds to six breeding rookeries from the same four islands but not including sites FD, IA, and PC (Figure 1A). Microsatellite genotyping was performed using 110 samples; 26 new samples collected in 2018 were analyzed with 84 samples from Lopes et al. (2015). The new samples were genotyped and calibrated locus-by-locus and a subset of samples from Lopes et al. (2015) (Supplementary Table S2). The samples obtained per rookery on each island were as follows: 1) Fernandina Island: Cabo Hammond (FH) (n = 26); 2) Isabela Island: Bahía Banks (IB) (n = 29), Cabo Marshall (IM) (n = 34), and Punta Flores (IF) (n = 7); 3) Pinta Island: Cabo Ibbetson (PI) (n = 5); and 4) Santiago Island: Puerto Egas (SE) (n = 9) (Figure 1A).

#### mtDNA Amplification, Sequencing, and Analysis

The mtDNA control region (CD) was partially amplified by PCR using the following primers: R3 (L15926) THR 5′-TCA​AAG​CTT​ACA​CCA​GTC​TTG​TAA​ACC-3 (Kocher et al., 1989) and TDKD (H16498) 5′-CCT​GAA​GTA​GGA​ACC​AGA​TG-3’ (Slade et al., 1994). Amplifications for CR were carried out in 50 μl with one unit of Platinum DNA *Taq* polymerase (Invitrogen), PCR buffer 1X (Invitrogen), 2.0 mM MgCl_2_, 0.25 mM dNTPs mix (Invitrogen), 0.5 μM of each primer, and 40 ng of template DNA. The thermocycling protocol consisted of an initial denaturation at 94°C for 5 min followed by 35 cycles of 94°C for 30 s, 65°C for 60 s, 72°C for 60 s, and one final cycle of 72°C for 7 min. Amplification products were visualized in 2% agarose (Promega) gel electrophoresis using SYBR safe (Invitrogen) for staining. The amplicons were further purified with the UltraClean PCR clean up kit, according to the manufacturer’s instructions. The purified products were sequenced in both directions using an ABI 3730XLs sequencer (Macrogen, Inc., Seoul, South Korea). The sequences were edited using PreGap4 and Gap4 from the Staden software package (Staden et al., 2000) to obtain consensus sequences using information obtained from sense and antisense DNA strands. The sequences were then aligned using MEGA X (Kumar et al., 2018) with ClustalW algorithm and trimmed to obtain sequences of equal lengths.

##### Population Diversity and Structure

Genetic diversity estimates were obtained using DNAsp v6.12.01 (Rozas et al., 2003). Haplotype diversity (*H*_*d*_), which is the measure of the uniqueness of particular haplotypes in a population, and nucleotide diversity (*π*), defined as the average number of differences per site between any two sequences chosen randomly from the sampled population, were assessed. Haplotype distribution and frequencies were obtained including their sequences and positions to construct a median-joining haplotype network (Bandelt et al., 1999) using Network v10.0 (Fluxus Technology Ltd.) (Bandelt et al., 1999). Genetic subdivision was estimated by calculating pairwise population’s *F*_ST_ (Weir and Cockerham, 1984) and via analysis of molecular variance (AMOVA) in ARLEQUIN 3.5.1.2 (Excoffier and Lischer, 2010). To estimate the amount of variation among and within rookeries, analyses were categorized on three different levels, by island (four groups), by rookery (nine sites for mtDNA), and by individuals. This analysis was carried out with 10,000 random permutations maximizing the examination of the variance explained among groups (four islands: *F*_CT_—values) and within populations, thus reflecting the strongest subdivisions. The components of covariance such as sigma (*σ*) and the percentage of variance were obtained for these three comparisons in both molecular datasets.

##### Genetic Bottlenecks and Inbreeding

Tajima’s *D* (Tajima, 1989) and Fu’s *Fs* (Fu, 1997) neutrality tests were used to infer population demographic changes (i.e., evidence for population expansions). The *Fs* values tend to be negative when there is an excess of recent mutations, and therefore statistically significant negative values provide evidence against population stasis (i.e., population growth and/or selection) (Fu, 1997).

#### Microsatellite Amplification, Genotyping, and Analyses

The primers for the eight loci used in this study were developed for Galapagos sea lion (*Zalophus wollebaeki:* ZcwB07, ZcwE04, ZcwF07, and ZcwE12) (Hoffman et al., 2007), gray seal (*Halichoerus grypus*: Hg8.10 and Hg6.3) (Allen et al., 1995), and harbor seal (*Phoca vitulina:* PvcE and Pv9) (Coltman et al., 1996) (Supplementary Table S2). The forward primers were 5′ tailed with the M13 sequence (5′-CAC​GAC​GTT​GTA​AAA​CGA​C-3′), and the M13 primer was marked with fluorophores FAM, HEX, or NED (Boutin-Ganache et al., 2001; Blacket et al., 2012). Amplifications were carried out in 10 µl with the following conditions: 1.5 mM MgCl_2_, 0.2 mM of each dNTP, 0.2 μM of reverse and M13-fluorescent primers, 0.133 μM of M13-tailed forward primer, 0.5 U of platinum Taq DNA polymerase (Invitrogen), 1X PCR buffer (Invitrogen), and 1 μl of DNA. The thermocycling conditions for the amplification were as follows: 2 min at 94°C; 40 cycles of 45 s at 94°C, 45 s at annealing temperatures described in the original references above, 50 s at 72°C; and a final extension of 2 min at 72°C. The PCR products were genotyped on an ABI 3730xl Capillary System at Macrogen Inc., Seoul, South Korea. The allele size (the number of bases) was identified and binned with the software Geneious V. 11.1.5 (Kearse et al., 2012). Micro-checker (Van Oosterhout et al., 2004) was used to test for the presence of genotype errors, evidence of null alleles, stuttering, and allele dropout in populations using an allelic matrix in the GENEPOP format.

##### Population Diversity and Structure

For each breeding population, exact tests were used to examine the deviation of each locus from the Hardy–Weinberg equilibrium using GENEPOP version 3.2a (Raymond and Rousset, 1995). A Bonferroni correction was applied on p-values to correct for multiple comparisons and minimize type I error (Rice, 1989). Genetic diversity indexes such as expected heterozygosity (*He*) and observed heterozygosity (*Ho*) for each locus and per population (breeding rookery) were calculated as implemented in *polysat* (Clark and Jasieniuk, 2011). Calculations of the number of private alleles (E) were performed in *proppr* (Kamvar et al., 2014), and the total number of alleles (A) across loci and individuals (K) in *adegenet* (Jombart, 2008) was calculated with software R (R Core Team, 2019). Population differentiation among rookeries was further explored using several approaches. First, genetic differentiation and pairwise *F*_ST_ values (Weir and Cockerham, 1984) and R_ST_ values were estimated in ARLEQUIN. We implemented an analysis of molecular variance AMOVA using *pegas* (Paradis, 2010) for software R (R Core Team, 2019), applying the same hierarchical ordination as previously used with the mtDNA dataset. In addition, the population structure was examined using STRUCTURE v2.3.4 (Pritchard et al., 2000), a Bayesian procedure to estimate the number of genetically distinct populations. The matrices for STRUCTURE were generated in PGD Spider v2.1.1.3 (Lischer and Excoffier, 2012), and the estimation of the optimal number of subpopulations (*K*) was completed using ten independent runs with *K* = 1–10 at 10^6^ MCMC generations combined with a 10^6^ burn-in period. Breeding rookeries were used as prior information in the admixture model (Hubisz et al., 2009), and correlated allele frequencies were implemented into the model (Falush et al., 2016), as recommended for small datasets (Janes et al., 2017). Rookery prior (LOCPRIOR) was used to explore the effect of philopatry in the population genetic structure by using sample group information (FH, IB, IF, IM, SE, and PI) in the clustering process. Given the nature of the study group (i.e., high vagility and thus expected low levels of divergence), this model was incorporated to assist in detecting the genetic structure but does not tend to find the structure where none is present (Pritchard et al., 2000). The optimal number of clusters (*K*) was obtained from ΔK by calculating the rate of change in the log probability of data between successive *K* values (Evanno et al., 2005) as implemented in the *ad hoc* approach, Structure Harvester online tool (Earl, 2012). Because uneven samples allocated to each rookery could lead to wrong inferences on the true number of subpopulations, the Puechmaille method was also applied (Puechmaille, 2016; Li and Liu, 2018). The threshold of the mean membership coefficient in *a priori* defined groups (here as six rookeries) is suggested to be set to 0.5 (or greater) and to consider *MedMeaK* or *MedMedK* instead of *MaxMedk* or *MaxMeanK* when selecting the clusters (Puechmaille, 2016). Therefore, we compared the results obtained from ΔK using the method recommended by Evanno et al. (2005) to clusters suggested from Puechmaille via StructureSelector (Li and Liu, 2018) using a threshold of 0.7. The consensus admixture plot from the 10 independent runs was obtained with the Clumpak tool (Kopelman et al., 2015).

##### Genetic Bottlenecks, Inbreeding, and Gene Flow

The possibility of recent population bottleneck was analyzed using BOTTLENECK (Piry et al., 1999) software. A 2-tailed Wilcoxon signed-rank test for heterozygosity excess (Luikart and Cornuet, 1998) was applied by detection of heterozygosity excess due to the rapid loss of low-frequency alleles and detection of small bottlenecks in natural populations with >5 polymorphic loci and >30 individuals. Excess of heterozygosity was examined using three models of mutation: stepwise mutation model (SMM, Ohta and Kimura, 1973), infinite allele model (IAM, Kimura and Crow, 1964), which are considered the two extreme models of mutation (Chakraborty and Jin, 1992), and the two-phase mutation model (TPM, Di Rienzo et al., 1994). Classically, allozyme data are assumed to fit the IAM model (Chakraborty, 1980), but most loci probably evolve according to a model intermediate between the SMM and IAM such as the TPM (Di Rienzo et al., 1994; Luikart and Cornuet, 1998). In addition, a qualitative descriptor of allele frequency distribution was used to infer bottlenecks via *F* (analogous to the inbreeding coefficient) (Jombart, 2008), defined as the probability for an individual to inherit two identical alleles from a single ancestor. This test relies on the fact that population bottlenecks cause a characteristic mode-shift distortion in the distribution of allele frequencies at selectively neutral loci. Bottlenecks cause alleles at low-frequency class (<0.1) to transitorily become less abundant than alleles in one or more intermediate allele frequency class, appropriate in analyses where sample size largely exceeds the minimum requirement of 30 specimens (Luikart and Cornuet, 1998). Thus, a test for mode shift in the expected distributions of allele frequencies from a normal L-shaped distribution to a mode-shift distortion is expected if populations have suffered a bottleneck. The application of this qualitative method was implemented in *adegenet* (Jombart, 2008) for software R (R Core Team, 2019) as a histogram of the frequency of *F* across all individuals. Alternatively, inbreeding coefficients *F*_IS_ (with 95% CI) were estimated using the *divbasic* function of the *diveRsity* package (Keenan et al., 2013) to explore changes in inbreeding across all rookeries. Finally, the microsatellite dataset was used to calculate the contemporary migration rates among islands (n = 6 rookeries) via the application divMigrate, allowing to resolve complex migration patterns (Sundqvist et al., 2016). We calculated the number of migrants (*Nm*) as a metric of connectivity between pairs of rookeries and the direction of gene flow as implemented in *diveRsity* (Keenan et al., 2013) for software R (R Core Team, 2019).

## Results

### Genetic Diversity

#### Mitochondrial DNA Control Region

Sequence analysis of the 219-bp mtDNA control region from 156 GFS individuals revealed 18 variable sites (all corresponded to transitions: Table 1 and Supplementary Table S2) among 21 unique haplotypes. The overall haplotype diversity and nucleotide diversities were 0.86 and 1%, respectively (Table 1). The highest haplotype diversity was observed at the Punta Flores rookery (IF) on Isabela Island (0.86) closely followed by Cabo Ibbetson (PI) on Pinta Island (0.85), with the lowest values at the Puerto Egas colony (SE) on Santiago Island (0.60). Santiago individuals also had the lowest nucleotide diversity (0.3%).

**TABLE 1:** Genetic diversity (*Hd* = haplotype diversity, π = nucleotide diversity and demographic parameters (Tajima’s D and Fu’s *F*_S_) estimated from the mtDNA control region sequences of Galapagos fur seal (*Arctocephalus galapagoensis*). N = number of samples; S = variable sites; H = total number of haplotypes; *uH* = number of unique haplotypes (not shared among sites); *Hd* = Haplotype diversity (**±** sd = standard deviation); and π = nucleotide diversity (**%**). Neither Tajima’s D nor Fu’s *F*_S_ resulted in significance (*p* < 0.05) for any population (actual p-values values not displayed).

Population	N	S	H	*uH*	*H*_*d*_ ± sd	π (%)	Tajima’s *D*	Fu’s *F*_*S*_
Overall	156	18	49	21	0.86 ± 0.017	1.2	−0.350	−5.843
Cabo Hammond (FH) Fernandina	44	13	11	6	0.77 ± 0.044	1.3	−0.129	−1.462
Cabo Douglas (FD) Fernandina	14	6	5	0	0.75 ± 0.084	1.0	0.842	0.458
Bahía Banks (IB) Isabela	30	5	6	2	0.77 ± 0.046	0.5	−0.263	−1.256
Punta Flores (IF) Isabela	6	7	4	0	0.86 ± 0.129	1.2	−0.630	−0.067
Punta Albemarle (IA) Isabela	10	5	5	1	0.75 ± 0.130	0.6	−0.682	−1.320
Cabo Marshall (IM) Isabela	31	7	6	1	0.80 ± 0.032	1.0	0.709	0.711
Puerto Egas (SE) Santiago	6	2	3	0	0.60 ± 0.215	0.3	−0.050	−5.843
Cabo Chalmers (PC) Pinta	7	4	4	0	0.71 ± 0.181	0.7	−0.039	−0.538
Cabo Ibbetson (PI) Pinta	8	5	5	1	0.85 ± 0.108	1.0	0.588	−0.965

#### Microsatellite Loci

Eight microsatellite loci were used to genotype 110 individuals, which revealed no evidence for amplification errors, null alleles, or allelic dropout. Linkage disequilibrium analyses revealed no association between markers (*p* > 0.05 after Bonferroni correction). In addition, none of the loci displayed significant deviations from the Hardy–Weinberg equilibrium; thus, all markers were considered for downstream genetic analyses. A total of 58 alleles were detected (mean = 7.25 alleles per locus), with only five private alleles in two breeding rookeries on Isabela Island (Bahía Banks IB; n = 2 and Cabo Marshall IM; n = 3) (Table 2). The number of alleles (Na) was the highest (Na = 48) in Bahía Banks (IB) on western Isabela Island and in Cabo Marshall (IM) (Na = 46) and Cabo Hammond (FH) (Na = 47) on Fernandina Island. The lowest number of alleles was found on Cabo Ibbetson (PI) (Na = 26) on the northeast island of Pinta. Microsatellite data revealed moderate genetic diversity with the expected heterozygosity (*He*) index of 0.69 and observed heterozygosity (*Ho*) of 0.66 (Table 2). The lack of significant difference between global *Ho* and *He* for all populations suggested a relatively negligible effect of inbreeding. The mean expected heterozygosity per rookery ranged from 0.70 in Cabo Marshall (IM) on Isabela Island to 0.61 on Puerto Egas (SE) on Santiago Island (average = 0.65), and the observed heterozygosity ranged from 0.71 in Cabo Marshall (IM) on Isabela Island to 0.51 in Cabo Ibbetson (PI) (average = 0.61) on Pinta Island.

**TABLE 2:** Genetic diversity indexes obtained for each rookery based on microsatellite data. N = number of individuals analyzed; Na = number of alleles; Np = private alleles; Ar = allelic richness; *He* = expected heterozygosity; *Ho* = observed heterozygosity; H-P = Hardy–Weinberg test of neutrality; and *F*_IS_ Inbreeding coefficient. Values in bold are significant (*p* < 0.001).

Population	N	(Na)	(Np)	Ar	(*He)*	(*Ho*)	H-P	*F*_IS_ (95% CI)
Cabo Hammond (FH)	26	47	0	3.87	0.67	0.67	0.104	−0.007 (−0.07,0.059)
Fernandina								
Bahía Banks (IB)	29	48	2	3.68	0.63	0.68	0.900	−0.09 (−0.157, −0.014)
Isabela								
Punta Flores (IF)	7	39	0	3.65	0.63	0.67	0.748	−0.055 (−0.244,0.081)
Isabela								
Cabo Marshall (IM)	34	46	3	3.87	0.70	0.71	0.136	−0.012 (−0.071,0.048)
Isabela								
Puerto Egas (SE)	9	37	0	3.39	0.61	0.53	0.416	0.125 (−0.5,0.264)
Santiago								
Cabo Ibbetson (PI)	5	26	0	2.79	0.69	0.51	0.374	0.35 (0.056,0.617)
Pinta								

#### Population Structure, Genetic Bottlenecks, and Gene Flow

##### Mitochondrial DNA Control Region

AMOVA analysis revealed that most of the variance was found within sampling localities (86.15%). However, the *F*_ST_ value (0.138) indicates that 13.8% of variance in haplotype frequencies is caused by the combined effects of island and rookery population subdivision (Table 3 and Supplementary Table S3). Despite the insignificant genetic structure found in mtDNA related to an island effect, the total number of haplotypes (H) was higher in Cabo Hammond (FH) (*H* = 11) on Fernandina Island, followed by Bahía Banks (IB) and Cabo Marshal (IM) (both values *H* = 6), followed by Cabo Douglas (FD), Punta Albermarle (IA) on Isabela Island, and Cabo Ibbetson (PI) on Pinta Island (all with values *H* = 5). Finally, Punta Flores (IF) on Isabela Island and Punta Chalmers on Pinta Island (PC) both had a value of *H* = 4, followed by Puerto Egas rookery (SE) on Santiago Island (*H* = 3) (Table 2). On a per island basis, the total number of haplotypes (*H*) was higher on Isabela with 21, followed by Fernandina with 16, Pinta with nine, and Santiago with three haplotypes. The number of unique haplotypes (*uH*) was higher on Fernandina with six, followed by Isabela with four, Pinta with one, and no unique haplotypes on Santiago. The haplotype network suggested a discernible haplotype differentiation with unique haplotypes located in rookeries on west Fernandina, in northwest locations on Isabela, and unique haplotypes found on the east on Santiago and Pinta islands toward the northeast of the archipelago. The rest of the haplotypes were shared among localities from Fernandina, Isabela, Santiago, and Pinta islands at the core of the network (most common haplotypes Ag3–Ag5).

**TABLE 3:** Analysis of molecular variance (AMOVA) using the mtDNA control region and eight microsatellite loci for *Arctocephalus galapagoensis*. Statistics for mtDNA: *F*_CT_ = 0.098, *F*_SC_ = 0.044, and *F*_ST_ = 0.138; Statistics for microsatellites *F*_CT_ = −0.011, *F*_SC_ = 0.037, and *F*_ST_ = 0.026. All values significant at *p* <0.001; df = degrees of freedom (Note: *F*-statistic estimators in the AMOVA are random variables and can take either positive or negative values. Such negative estimates should be interpreted as zero in the AMOVA (Schneider et al., 2000)).

	mtDNA		Microsatellites	
Genetic differentiation	Variance (σ)	% of variation	Variance (σ)	% of variation
Among groups (islands) df = 3	0.0445	9.85	0.0045	0.15
Among populations (rookeries) within groups (islands) (df = G-P; 9–5 =5)	0.0181	4.00	0.2106	6.86
Within populations (among individuals within rockeries)	0.3891	86.15	2.8565	92.99

##### Microsatellite Loci

No significant island differentiation was detected by AMOVA analyses among the GFS breeding colonies (93% within sampling localities) with little variance found within rookeries (2.56%) for microsatellite markers (Table 3). However, the STRUCTURE analysis with the ΔK method suggested two clusters (*K* = 2), whereas the estimators *MedMeaK* or *MedMedK* suggested three genetic clusters (*K* = 3). In both results, the first group includes all samples from western localities on Fernandina (Cabo Hammond (FH)) and Isabela islands (Bahía Banks (IB)). The second genetic cluster recovered from the ΔK method corresponded to the remainder of the samples: north and northeast of Isabela (Cabo Marshall (IM) and Punta Flores (IF)), Santiago (Puerto Egas (SE), and the northeast island of Pinta (Cabo Ibbetson (PI)). The Puechmille method using the *MedMeaK* and *MedMedK* estimators pulled Pinta individuals from this second cluster and placed them into a third independent population (*K* = 3; Figures 1D and Supplementary Figure S1).

##### Genetic Bottlenecks, Inbreeding, and Gene Flow (Both Datasets)

Three populations showed significant heterozygosity excess (based on the Wilcoxon signed-rank test under the IAM model) as a consequence of bottleneck events: Cabo Ibbetson (PI), Cabo Marshall (IM), and Cabo Hammond (FH). With the TPM model, only two populations showed significant heterozygosity excess (Cabo Ibbetson (PI) and Cabo Marshall (IM)), and with the SSM model, only Cabo Ibbetson (PI) (Supplementary Table S4). Population bottleneck and its impact on levels of inbreeding were visually confirmed by the high frequency of low *F* values class (<0.1), producing a mode-shift distortion in the typical L-shaped distribution of allele frequencies (Supplementary Figure S2). Furthermore, inbreeding coefficients *F*_IS_ showed larger values at Cabo Ibbetson (PI; *F*_IS_ = 0.35) on Pinta Island and Puerto Egas (SE; *F*_IS_ = 0.125) on Santiago Island, with the lowest values on Bahía Banks (IB; *F*_IS_ = −0.009) and Cabo Hammond (FH; *F*_IS_ = −0.007) on and western Isabela and Fernandina Islands, respectively (Table 2, Supplementary Figure S3). The test for neutrality did not report a significant F*s* value (Fu and Li, 1993) or deviation from neutrality (Tajima, 1989) on any of the studied populations (Table 1).

Using eight microsatellite loci, historical gene flow and its directionality were determined to be predominantly from Bahía Banks (IB) on Isabela Island to Cabo Hammond (FH) on Fernandina Island (*Nm* = 1) and only half of its strength in the opposite direction (*Nm* = 0.5). Migration between the rest of the rookeries was moderately low. Particularly, the Cabo Ibbetson colony on Pinta Island appears to be the most isolated regarding the incoming gene flow from any other rookeries and contributing with a low number of migrants to the rest of the colonies (Figure 1C and Supplementary Figure S4).

## Discussion

Our results indicated the existence of at least two major genetic groups of GFS across its entire breeding range on the Galapagos archipelago. These findings are in contrast to the genetic structuring and geographic population differentiation found by Lopes et al. (2015). This discrepancy is mostly due to the increased geographic sampling of key new rookeries (Santiago and Pinta islands). Interestingly, population genetic metrics (*Hd* and π) for both markers did not vary between these two studies, although direct comparison is made difficult given the uneven number of loci genotyped [eight microsatellites here; 18 microsatellites in the study by Lopes et al. (2015)]. On the other hand, the discernible contribution of additional rookeries to genetic structuring could be explained by the detection of new haplotypes and dissimilar allele frequencies found at some rookeries. For instance, new and endemic haplotypes on Pinta (Ag17 and Ag18) are reported for the first time, and haplotypes previously found on Isabela and Fernandina were also recovered on newly sampled Santiago and Pinta islands (Ag03, Ag11, and Ag12). Allele frequencies were also found to be similar between the east Isabela sites and Santiago rookery, whereas Pinta seems to be an admixture of the other rookeries as shown in the Puechmille method analysis.

In general, genetic structure in pinnipeds can be shaped by several factors including geographic barriers (vicariance in refugia: Bickham et al., 1998), climatic events such as El Niño–Southern Oscillation (ENSO) (Goldsworthy et al., 2000; De Oliveira et al., 2009), sex-biased dispersal and philopatry (Nyakaana and Arctander, 1999; Escorza-Treviño and Dizon, 2000), and even historical hunting by humans. Furthermore, previous studies on Galapagos biota support the one-island-one species model [e.g., marine iguanas (Steinfartz et al., 2009) and lava lizards (Benavides et al., 2009)] resulting from limited dispersal and long periods in isolation. This model, although possible in pinnipeds (Bickham et al., 1998), and thus probable in GFS, does not concur with the phylogeographic pattern obtained here: shared mtDNA haplotypes across islands. In addition, human presence on the archipelago has had a strong influence on its biota. Population declines in GFS by hunting have been reported in the past with potential effect on its population dynamics (Folkens and Reeves, 2002). In addition, climatic fluctuations such as ENSO and its effects on marine productivity have also caused dramatic declines in GFS populations in the last four decades (Páez-Rosas et al., 2021). However, our bottleneck analysis does not support a strong effect on the genetic diversity of GFS populations related to these factors. That is, the oscillations in effective population sizes were negligible as suggested by the high levels of nucleotide and haplotype diversity recovered here and by Lopes et al. (2015). This pattern could correspond to the persistence of abundant populations escaping hunting pressures on inaccessible shores or islands (Stoffel et al., 2018). However, we do not discard the demographic decline effects that both ENSO events and hunting have had in the abundance of Galapagos otariids in the past (Riofrío-Lazo and Páez-Rosas, 2021).

In insular systems, the population structure could also be attributable to philopatry and limited dispersal, although this might seem unlikely in a highly mobile species, such as GFS (Villegas-Amtmann et al., 2013). Our findings suggest a strong separation of populations, and the genetic clusters recovered (either *K* = 2 or *K* = 3) corresponded to 1) individuals from Cabo Hammond (FH) on Fernandina Island and Bahía Banks (IB) on west Isabela Island at the westernmost distribution; 2) individuals from Cabo Marshall (IM) and Punta Flores (IF) from the north and east of Isabela Island and individuals from Puerto Egas (SE) on Santiago Island; and 3) individuals from Cabo Ibbetson (PI) on Pinta Island clustering weakly with cluster 2 but potentially establishing its own unique genetic cluster at the northeastern tip of the distribution with contributions from both previous clusters. Interestingly, we found a marked genetic differentiation between rookeries found on the same island (Isabela), separated by less than 10 km [Bahía Banks (IB) and Punta Flores (IF)], whereas strong genetic connectivity was found between IB and rookeries from the neighboring Fernandina Island (Cabo Hammond (FH)) over 25 km apart. It is possible that despite having the ability to move long distances, GFS present signals of restricted gene flow, probably related to site fidelity not only by females but also by males as indicated by mtDNA and nuDNA results.

Philopatry is usually marked in female Galapagos otariids (Trillmich and Wolf, 2008; Wolf et al., 2008), but our estimates of gene flow for both sexes show that males also tend to stay in the same genetic cluster, contributing to the reinforcement of population structure. The fact that nuclear biparentally inherited markers tend to take longer to show patterns of subdivision than maternally inherited ones (four times faster in mtDNA) (Moritz, 1994) suggests that males also show significant philopatry over a sufficiently long time. Gene flow analyses showed directional movement of migrants from Bahía Banks (IB) on Isabela to Cabo Hammond (FH) on Fernandina (number of migrants *Nm* = 1), but only half of its strength in the opposite direction (*Nm* = 0.56). If these movements were mostly carried out by males, they present a relative higher contribution of genes toward Fernandina rookeries. This phenomenon is also evident in the maternally inherited mtDNA with a higher number of endemic haplotypes from Fernandina. A large number of haplotypes corresponded to rookeries separated by hundreds of kilometers, including four haplotypes also found in Fernandina, suggesting some “leakage” from Fernandina to the rest of rookeries but not in the opposite direction. The presence of unique and shared haplotypes on Pinta can be indicative of asymmetric connectivity with several rookeries on other islands. This result was supported by low levels of gene flow from Pinta to other rookeries and almost negligible on the other directions. Given the high fidelity of females, this level of haplotype sharing might correspond to haplotypes found in males that have migrated from Fernandina to mate somewhere else. As mentioned before, such sex-bias behavior is commonly reported in other otariids (e.g., Campbell et al., 2008) and could explain the genetic signatures presented here.

An emerging question from this pattern is: what conditions affect such strong natal habitat preference in both sexes that could reinforce the levels of genetic differentiation found in both genetic datasets? In this context, local productivity seems a plausible putative explanation. The Galapagos Islands are characterized by different levels of marine productivity, with higher values on the western region resulting from the upwelling of the nutrient-rich Cromwell current, trade winds, and equatorial substream flows (Pak and Zaneveld, 1973; Palacios et al., 2006; Schaeffer et al., 2008). The plume with elevated chlorophyll levels on the west side of Isabela and Fernandina islands is responsible for the high densities of marine mammals tightly linked to the reproductive success of several endemic species, including the Galapagos pinnipeds (Wolf et al., 2008; Alava, 2009). It has been suggested that such hotspots deeply affect genetic differentiation via strong habitat preferences in species associated with this abundance of food resources [e.g., Galapagos sea lions (Wolf et al., 2008) and marine iguanas (Steinfartz et al., 2009)]. In Galapagos sea lions (GSLs) more specifically, hotspot preferences combined with a strong “social environment” (i.e., socially mediated natal habitat learning) (Wolf et al., 2008), could explain the reported genetic groups associated with these rich and deep waters on the western portions of Isabela and Fernandina. Female GSL, particularly before pupping and during lactation, tend to choose high productivity areas to reduce time spent on foraging trips (Merrick and Loughlin, 1997). Strong bonds between adults and young individuals in social animals could facilitate learned feeding niches or habitat selection (Slagsvold and Wiebe, 2007), which is evidenced in foraging philopatry in both females and males in Galapagos otariids in the western side of the archipelago (Páez-Rosas et al., 2012; Drago et al., 2016).

Mean distances from rookeries to food patches might also impact local feeding adaptation and inherited feeding strategies. For instance, the maternal care strategy of GFS involves cycles of nursing interspersed with short periods at sea (17.9 ± 10.6 h), before returning to feed the pup (Trillmich, 1987; Villegas-Amtmann et al., 2013). Weaning in GFS occurs about 18–36 months after birth, depending on environmental conditions (Trillmich and Wolf, 2008). This long investment of mothers limits their mobility and could reinforce the philopatry of females. Although long-term fidelity has been shown in GSLs to remain for several years, maternal home range inheritance by immature females could also affect genetic structuring within a breeding colony via genetic relatedness and inclusive fitness benefits (Moses and Millar, 1994; Wolf and Trillmich, 2007). Similarly, high philopatry in males could result in year-round density-dependent elements when establishing or defending valuable territories. Thus, highly productive foraging grounds could drive male philopatry due to the need for short and nearby foraging trips that allow them to be able to defend high-quality territories. The unusually prolonged breeding season in GFS compared to other fur seal species (Trillmich, 1987) could contribute to male and female philopatry, thus affecting the genetic differentiation between high- and low-productivity regions. But, most of these factors do not explain the genetic composition of rookeries on the north of Isabela, Santiago, or on Pinta where upwelling conditions are less marked.

The concentration of GFS populations on the westernmost island of the archipelago is as much as 61% of the entire population on two rookeries (Cabo Hammond (FH) and Cabo Douglas (FD), Fernandina Island) (Páez-Rosas et al., 2021). These percentages are disproportionately high compared to those reported in Pinta (12.7%) and northern Isabela (10.4%) populations. The population of Santiago Island is very small (3.2%) (SE = 91) and has been categorized as a recolonization that is in the process of growth (Páez-Rosas et al., 2021). This asymmetric distribution pattern is common in other marine species that also have their largest populations along the western archipelago, putatively due to the high productivity of this region [marine iguanas (Steinfartz et al., 2009); Galapagos penguin (Vargas et al., 2005)]. Dispersal movements to extreme eastern rookeries on Pinta, on the other hand, could be motivated by density-dependent factors to escape from crowding and local competition as reported in other pinnipeds (O’Corry-Crowe et al., 2014). That is, rookeries close to the productive foraging areas might contribute migrants to distant rookeries, suggesting that islands such as Pinta may be suboptimal habitats for GFS. Pinta was the only rookery to show significant signatures of population expansion (Fu’s *F*) as a result of a recent founder event explaining its separation in a third genetic cluster. These results are corroborated with the most significant increase in population numbers from 40 individuals reported in 2001 (Alava and Salazar 2006) to an average of 313 individuals counted in 2014–18 (Páez-Rosas et al., 2021), making it the third largest rookery to date. Further gene flow analysis could reveal the origin of this rookery.

Generally, species that can persist in refuges and have the capability to disperse are the most resistant to anthropogenic and environmental disturbances (Pinsky et al., 2010). Our results suggest that GFS is found in a dynamic productivity hotspot which could pose some unpredicted consequences in the face of refugia disturbance. It is important to note that despite the protection granted by the Galapagos Marine Reserve, this endemic species is not free from regional-scale perturbations of highly productive waters. The direct threat of competition and pollution generated by the eastern Pacific industrial tuna fleet, which concentrates its fishing activity in this region (Bucaram et al., 2018; Ventura et al., 2019), and the projected severe climate extremes menacing the stability of this system (Wang et al., 2017; Cai et al., 2018; Forryan et al., 2021) are stark threats to the long-term persistence of this endangered species, given its high reliance on local upwelling. Here, we propose that the elevated marine productivity in the west of the archipelago represents areas of high-quality habitat selected by individuals across several generations, reinforcing the observed patterns of genetic differentiation in GFS. This strong association could result in widespread patterns of population genetic structure in other marine species distributed along this productivity gradient in the Galapagos archipelago.

## Data Availability

The data presented in this article can be found in GenBank: MZ419385-MZ419391. Additional datasets can be found at https://doi.org/10.6084/m9.figshare.19620564.
