# Supplementary material for: Population Genetics and Phylogeography of Galapagos Fur Seals
Source: Front Genet. 2022 May 19;13:725772. doi: 10.3389/fgene.2022.725772 (PMC9160918; doi:10.3389/fgene.2022.725772)
Supplement: Supplementary file 1 [file DataSheet1.docx]

**SUPPLEMENTARY INFORMATION**

**Table S1**: Mitochondrial haplotypes and polymorphic sites.

| Haplotype | Accession | Polymorphic sites | | | | | | | | | | | | | | | | | | Frequency | Individuals | Reference |
| --- | --- | --- | --- | --- | --- | --- | --- | --- | --- | --- | --- | --- | --- | --- | --- | --- | --- | --- | --- | --- | --- | --- |
|  |  | 53 | 73 | 80 | 91 | 107 | 136 | 137 | 155 | 159 | 169 | 161 | 171 | 191 | 197 | 200 | 209 | 212 | 214 |  |  |  |
| Ag1 | KM030335 | G | C | G | G | A | T | T | T | T | G | A | G | T | C | C | C | A | G | 21 | FH01 FH06 FH07 FH08 FH15 FH17 FH24 FH25 FH27 FH28 FH30 **FD07 FD08 FD11 FD12** **FH41** **FH42** **FH43** **FH45** **FH47** **FH55** | Lopes *et al*., 2015 |
| Ag2 | KM030336 | . | . | A | . | G | . | . | C | . | . | . | . | . | . | . | . | . | . | 6 | FH02 FH09 FH18 FH23 FH29 **FH50** | Lopes *et al*., 2015 |
| Ag3 | KM030337 | . | . | A | . | . | . | C | . | C | . | . | A | . | . | . | . | . | . | 46 | FH03 FH05 FH11 FH14 FH16 FH20 FH21 FH22 FH26 IB07 IB12 IB13 IB16 IB17 IB22 IB23 IB26 IB27 IB29 IM01 IM19 IM23 IM24 IM25 IM28 **IA01 IA03 IA05 IA10 IA12** **FD01 FD03 FD04 FD06 FD14 FD15 PI03 PI04 IF24 IM39 IM41** **FH46** **FH48** **FH52** **PC02** **SE02** | Lopes *et al*., 2015 |
| Ag4 | KM030338 | . | T | . | . | . | . | . | . | . | . | . | . | . | . | . | . | . | . | 1 | FH04 | Lopes *et al*., 2015 |
| Ag5 | KM030344 | A | . | A | . | . | . | C | . | C | . | . | A | . | . | . | . | . | . | 15 | FH10 IB05 IB19 IM03 IM08 IM16 IM17 IM18 IM20 **IA04 IA08** **FD05** **IF25 IF26** **SE09** | Lopes *et al*., 2015 |
| Ag6 | KM030346 | . | . | A | . | . | . | C | . | C | . | . | A | . | T | . | . | . | . | 14 | FH12 FH19 IB01 IB03 IB04 IB06 IB11 IB15 IB18 IB20 IB24 IB25 **FD02 FD09** | Lopes *et al*., 2015 |
| Ag7 | KM030347 | . | . | A | . | G | . | . | . | . | . | . | . | . | . | . | . | . | . | 1 | FH13 | Lopes *et al*., 2015 |
| Ag8 | KM030366 | . | . | A | . | . | . | C | . | C | . | G | A | . | T | . | . | . | . | 5 | IB02 IB08 IB09 IB28 **IA06** | Lopes *et al*., 2015 |
| Ag9 | KM030374 | . | T | A | . | . | . | C | . | C | . | . | . | . | . | . | . | . | . | 2 | IB10 IB21 | Lopes *et al*., 2015 |
| Ag10 | KM030394 | . | . | A | . | . | . | C | . | C | . | . | A | . | T | . | T | . | . | 2 | IB14 IB30 | Lopes *et al*., 2015 |
| Ag11 | KM030396 | . | . | A | A | . | . | C | . | C | . | . | A | . | . | . | . | . | . | 15 | IM02 IM04 IM09 IM12 IM13 IM14 IM21 IM22 **PI01 IM40 PC01 SE03 SE04 SE08 SE10** | Lopes *et al*., 2015 |
| Ag12 | KM030400 | . | . | A | . | . | . | C | . | C | . | . | A | C | . | . | . | . | . | 12 | IM05 IM06 **IA09** **PI02 PI07 PI08 IF21 IF22 PC03 PC04 PC05 PC07** | Lopes *et al*., 2015 |
| Ag13 | KM030401 | . | . | A | A | . | . | C | . | . | A | . | . | . | . | . | . | G | . | 6 | IM07 IM10 IM11 IM15 **IF23 IM43** | Lopes *et al*., 2015 |
| Ag14 | KM030421 | . | . | A | . | . | . | C | . | C | . | . | A | C | . | . | . | G | . | 1 | IM27 | Lopes *et al*., 2015 |
| **Ag15** |  | . | . | A | . | . | . | C | . | . | . | G | A | . | T | . | . | . | . | 1 | **IA11** | This study |
| **Ag16** |  | . | . | A | . | . | . | . | . | . | . | . | . | . | . | . | . | . | . | 2 | **FD13 FH53** | This study |
| **Ag17** |  | . | . | A | A | . | . | C | . | C | . | . | A | . | T | . | . | . | A | 2 | **PI05 PC06** | This study |
| **Ag18** |  | . | . | A | A | . | . | C | . | . | . | . | A | . | T | . | . | . | A | 1 | **PI06** | This study |
| **Ag19** |  | . | . | . | . | . | . | . | . | . | . | . | . | . | . | T | . | . | . | 1 | **FH44** | This study |
| **Ag20** |  | . | . | A | . | G | . | . | C | . | . | . | . | . | . | . | T | . | . | 1 | **FH49** | This study |
| **Ag21** |  | . | . | A | A | . | C | C | . | C | . | . | A | . | . | . | . | . | . | 1 | **FH54** | This study |

**Table S2**. SSR markers description. Name maker, dye used, size, alleles per locus, expected (*He*) and observed (*Ho*) heterozygosity, and Hardy-Weinberg test of neutrality p-values.

| **Maker** | **Dye** | **Observed Size** | **Alleles per locus** | **Expected heterozygosity (*He)*** | **Observed heterozygosity (*Ho*)** | **Hardy-Weinberg test p-value** |
| --- | --- | --- | --- | --- | --- | --- |
| ZcwE12 | Hex | 173-187 | 7 | 0.77 | 0.72 | 0.828 |
| Hg 8.10 | Ned | 178-188 | 6 | 0.63 | 0.54 | 0.133 |
| PvcE | 6-Fam | 118-138 | 8 | 0.67 | 0.53 | 0.984 |
| Pv9 | Hex | 172-182 | 6 | 0.72 | 0.72 | 0.489 |
| Hg 6.3 | Ned | 225-239 | 6 | 0.57 | 0.53 | 0.483 |
| ZcwE04 | 6-Fam | 120-144 | 10 | 0.86 | 0.90 | 0.292 |
| ZcwF07 | Hex | 146-162 | 6 | 0.51 | 0.53 | 0.751 |
| ZcwB07 | Ned | 182-198 | 9 | 0.80 | 0.84 | 0.329 |
| **Global** | **-** | **-** | **7.25** | **0.69** | **0.66** | **-** |

**Table S3**: Estimated pairwise values of *F*_ST_ from mtDNA control region. Significant values (*p* < 0.05) were tested with confidence intervals by 1,000 Bootstrap replicates. No significant pairwise were obtained. Rookeries: FH (Cabo Hammond-Fernandina), FD (Cabo Douglas-Fernandina), IB (Bahía Banks-Isabela), IF (Punta Flores-Isabela), IA (Punta Albemarle-Isabela), IM (Cabo Marshall-Isabela), SE (Puerto Egas-Santiago), PC (Cabo Charlmers-Pinta), PI (Cabo Ibbetson-Pinta).

|  | **FH** | **FD** | **IB** | **IF** | **IA** | **IM** | **SE** | **PC** | **PI** |
| --- | --- | --- | --- | --- | --- | --- | --- | --- | --- |
| **FH** | - |  |  |  |  |  |  |  |  |
| **FD** | 0.06777 | - |  |  |  |  |  |  |  |
| **IB** | 0.43385 | 0.27230 | - |  |  |  |  |  |  |
| **IF** | 0.27901 | 0.10829 | 0.25916 | - |  |  |  |  |  |
| **IA** | 0.32975 | 0.13300 | 0.06242 | -0.05495 | - |  |  |  |  |
| **IM** | 0.33959 | 0.17879 | 0.26507 | 0.04984 | 0.10019 | - |  |  |  |
| **SE** | 0.38299 | 0.26942 | 0.37342 | 0.03480 | 0.23212 | -0.01825 | - |  |  |
| **PC** | 0.38033 | 0.24207 | 0.29215 | 0.04784 | 0.13187 | 0.10421 | 0.21153 | - |  |
| **PI** | 0.34904 | 0.18806 | 0.21358 | 0.02231 | 0.08342 | 0.06494 | 0.08730 | -0.09841 | - |

**Table S4.** Wilcoxon test (*p*-values) for heterozygosity excess or deficiency for IAM, TPM and SSM models. Numbers in bold significant (p < 0.001).

| **Population** | **IAM** | **TPM** | **SSM** |
| --- | --- | --- | --- |
| Cabo Hammond (FH)* | **0.0039** | 0.054 | 0.546 |
| Bahía Banks (IB)** | 0.4609 | 0.640 | 0.039 |
| Punta Flores (IF)** | 0.5468 | 0.945 | 0.843 |
| Cabo Marshall (IM)** | **0.0039** | **0.007** | 0.546 |
| Puerto Egas (SE) *** | 0.7421 | 0.640 | 0.546 |
| Cabo Ibbetson (PI) **** | **0.0078** | **0.007** | **0.007** |

*Fernandina, **Isabela, ***Santiago, ****Pinta


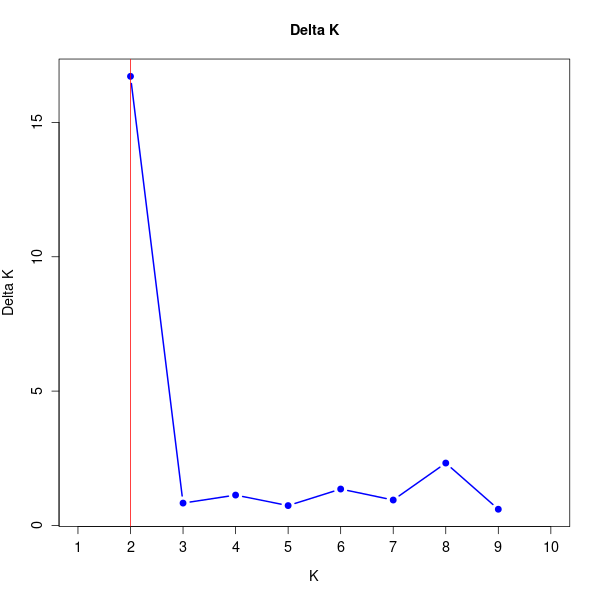

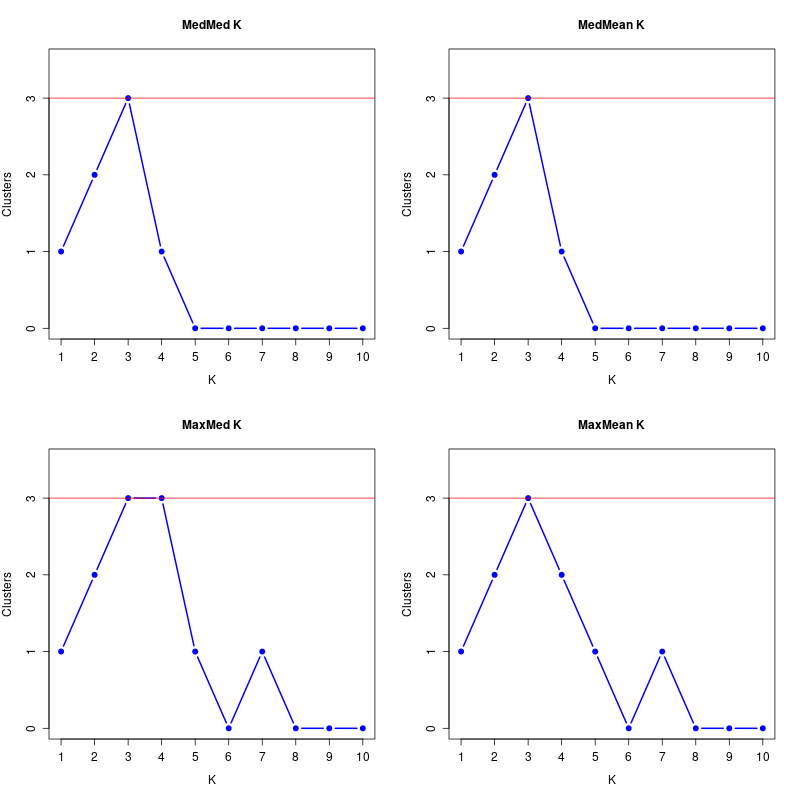


**Figure S1**. Bayesian clustering analysis ΔK (left) (Evanno et al., 2005) showing best *K*=2 and Puechmaille Method using a threshold = 0.7. Above panels showing best *K*=3 after MedMedK and MedMeanK models. Below panels showing alternative clusters *K*= 3-4 suggested for MaxMedK and *K*=3 for MaxMeanK.


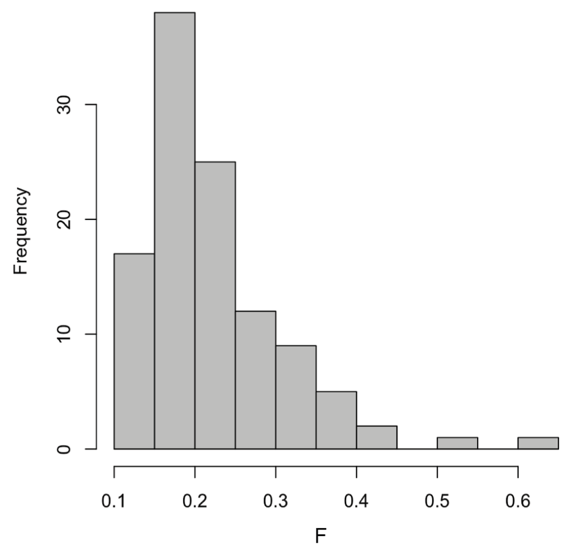


**Figure S2.** Inbreeding coefficient (*F*) histogram of *Arctocephalus galapagoensis* from 110 individuals from all the islands combined. Distribution skewed to the left (= high frequency of small *F* values) suggesting rare inheritance of homozygote genotypes from inbreeding.


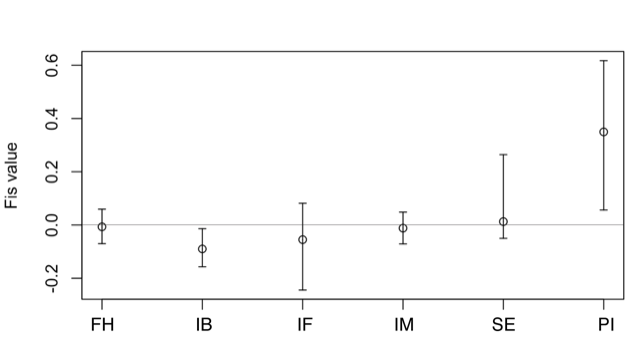


**Figure S3.** Fixation index (*F*_IS_) estimated values from eight SSR markers and 95% confidence intervals based on 10.000 bootstrap values for *Arctocephalus galapagoensis* rookeries. Locations: FH (Cabo Hammond), IB (Bahía Banks), IF (Punta Flores), IM (Cabo Marshall), SE (Puerto Egas), PI (Cabo Ibbetson).

**
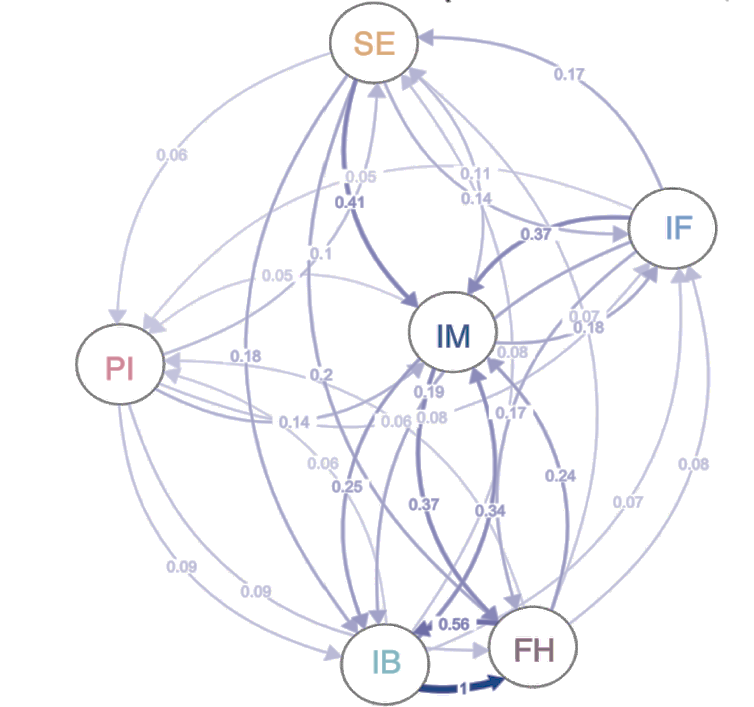
**

**Figure S4.** Relative migration network and directionality based on eight SSR markers. Circles represent sampling sites, arrows mark the direction of gene flow, and thickness is proportional to the level of migrant exchange between locations (numbers migrants *Nm*). Rookeries: FH (Cabo Hammond-Fernandina), IB (Bahía Banks-Isabela), IF (Punta Flores-Isabela), IM (Cabo Marshall-Isabela), SE (Puerto Egas-Santiago), PI (Cabo Ibbetson-Pinta).
